# Supplementary figures and images for: Saponin Improves Recovery of Bacteria from Orthopaedic Implants for Enhanced Diagnosis Ex Vivo
Source: Microorganisms. 2025 Apr 7;13(4):836. doi: 10.3390/microorganisms13040836 (PMC12029792; doi:10.3390/microorganisms13040836)

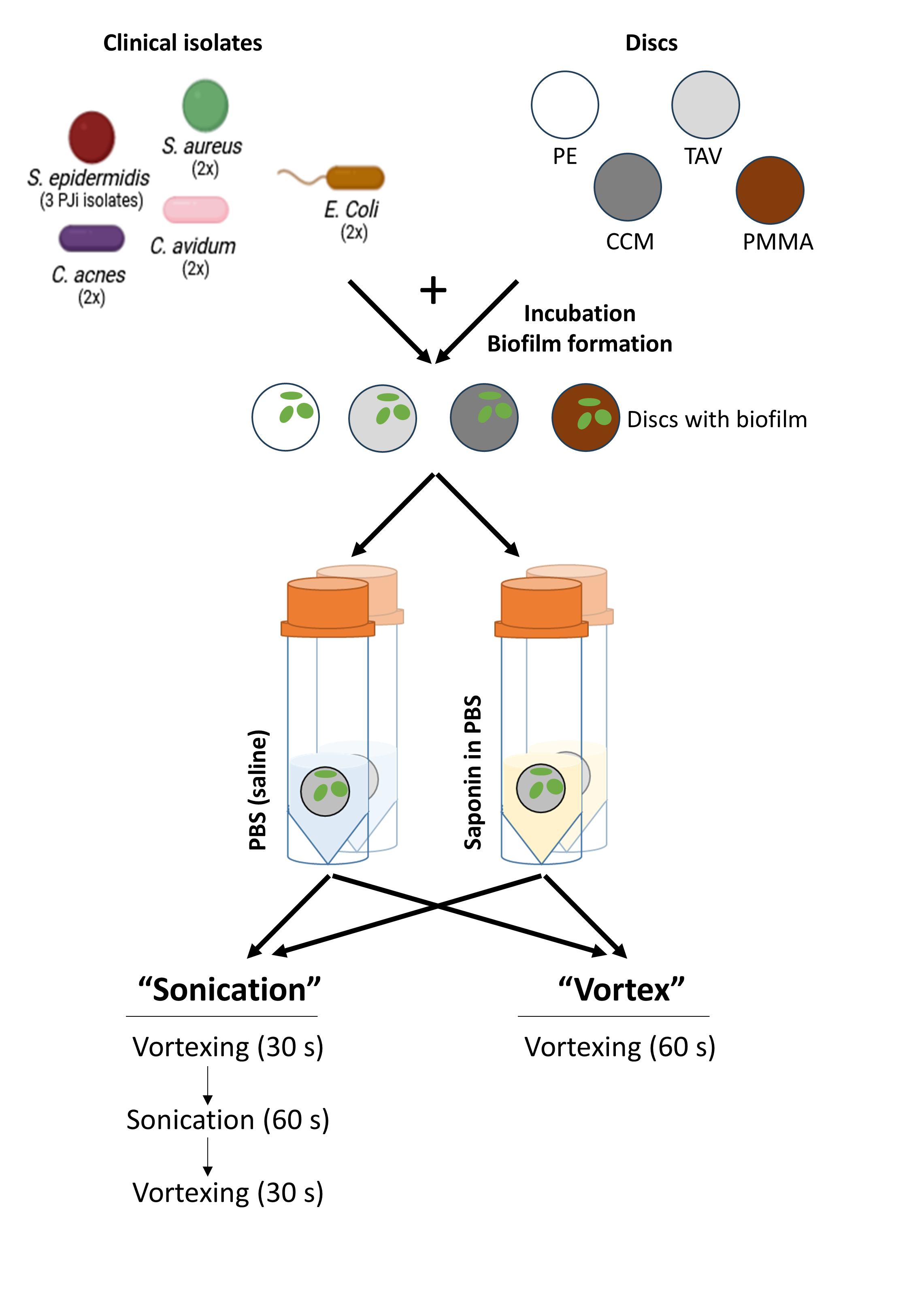

Supplement: Supplementary file 1 [file microorganisms-13-00836-s001.zip › Suppl Fig 1.tif]

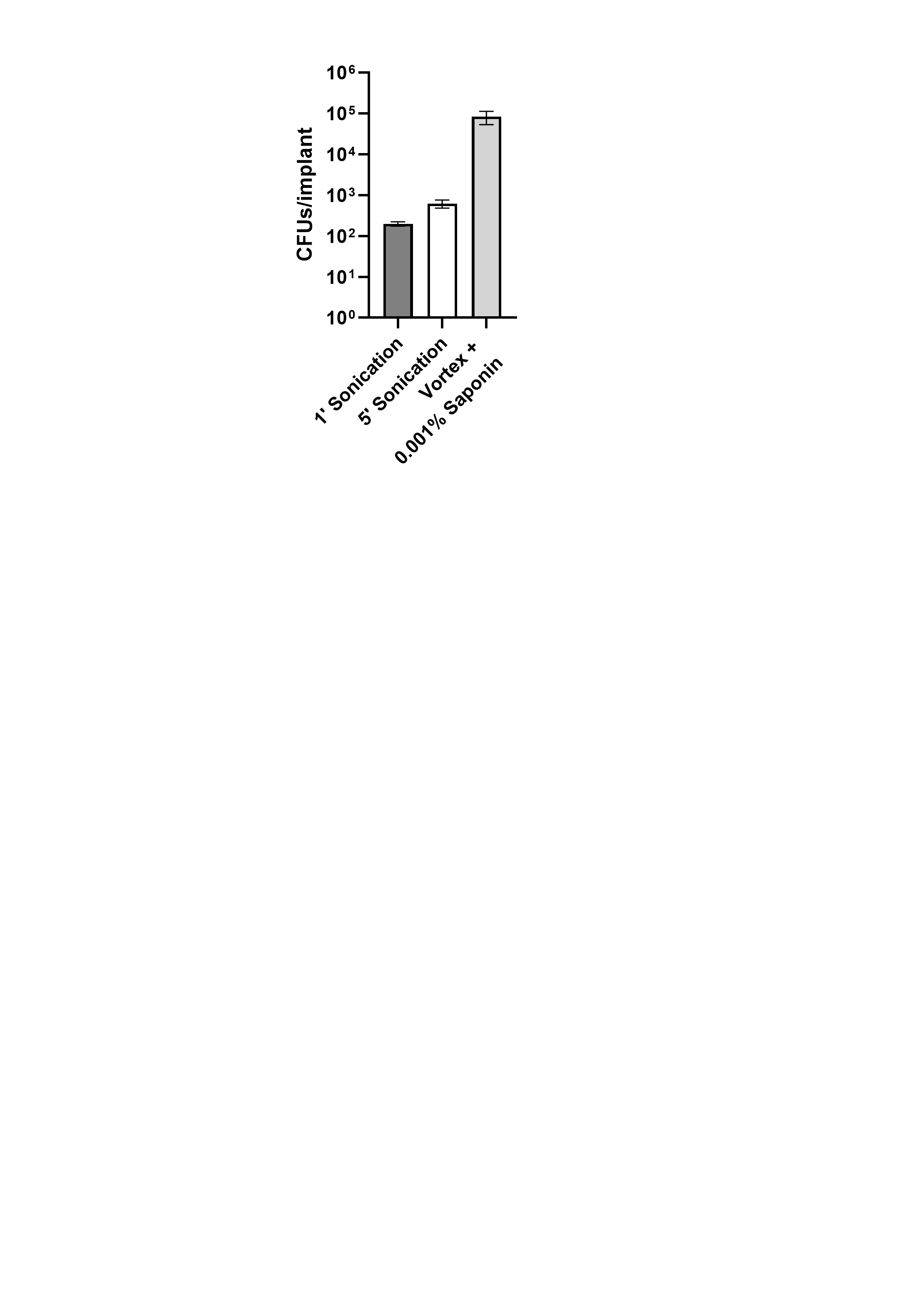

Supplement: Supplementary file 1 [file microorganisms-13-00836-s001.zip › Suppl Fig 2.TIF]

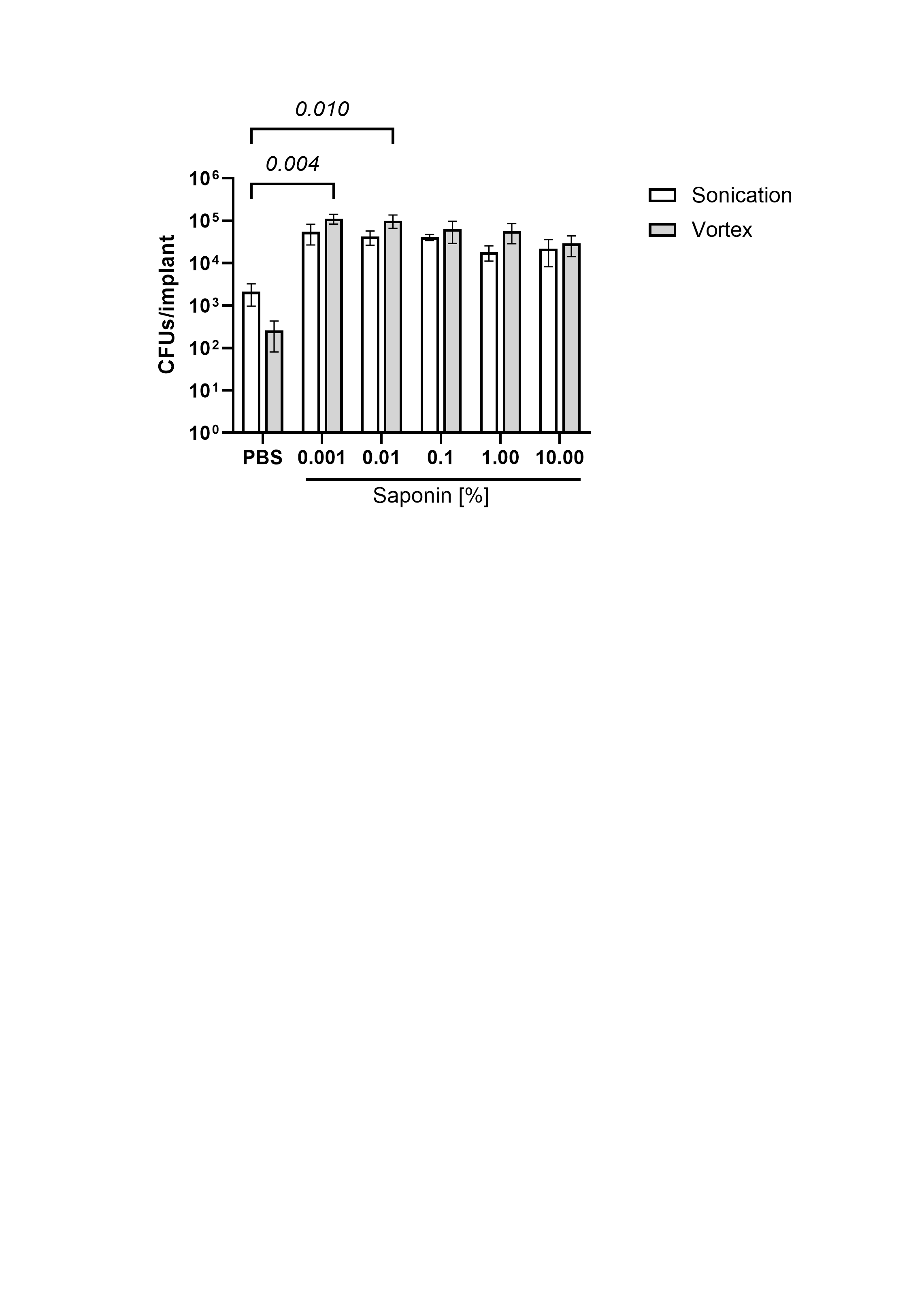

Supplement: Supplementary file 1 [file microorganisms-13-00836-s001.zip › Suppl Fig 3.TIF]

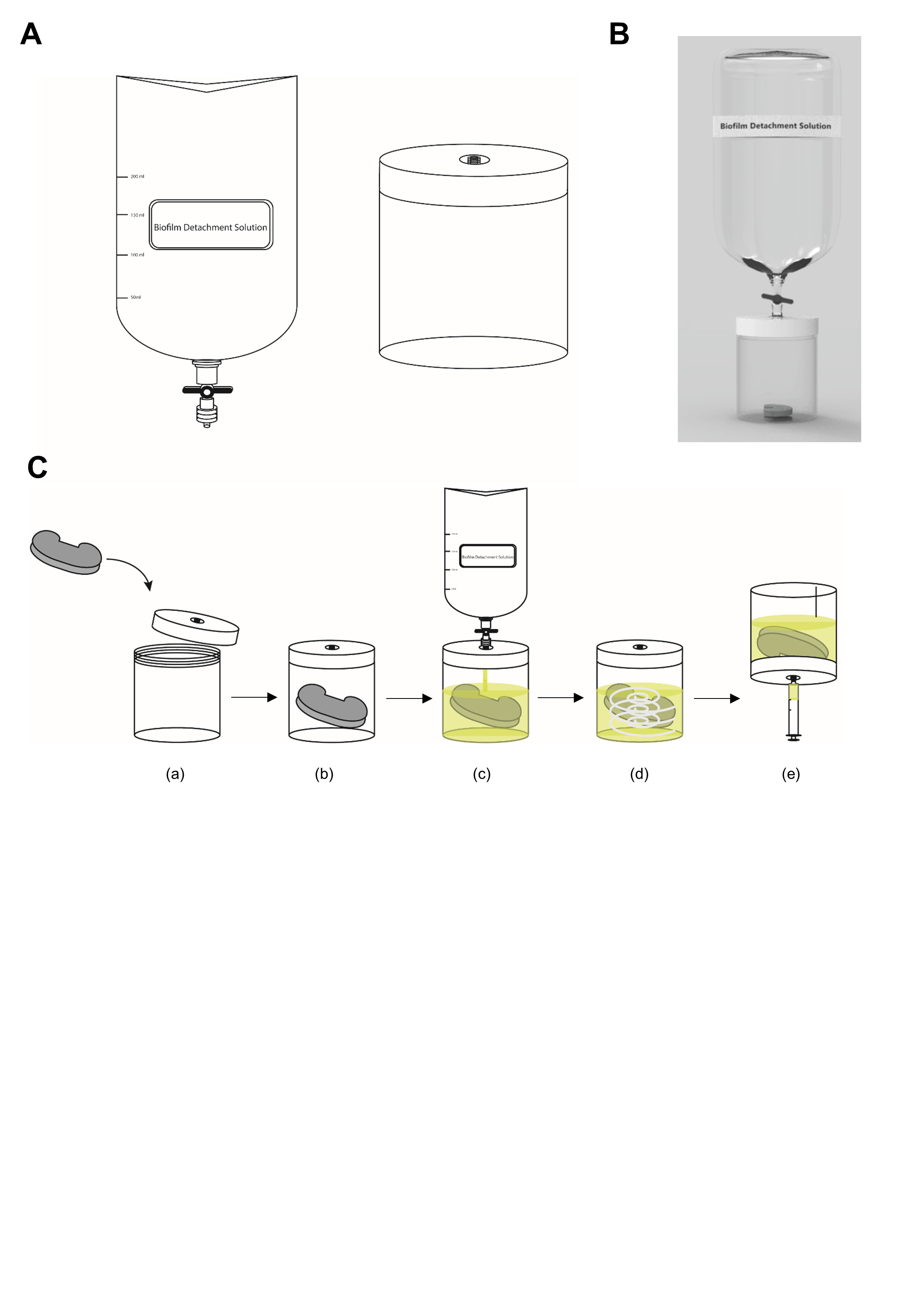

Supplement: Supplementary file 1 [file microorganisms-13-00836-s001.zip › Suppl Fig 4.TIF]
